# A sport-specific wearable jump monitor for figure skating

**DOI:** 10.1371/journal.pone.0206162

**Published:** 2018-11-21

**Authors:** Dustin A. Bruening, Riley E. Reynolds, Chris W. Adair, Peter Zapalo, Sarah T. Ridge

**Affiliations:** 1 Exercise Sciences Department, Brigham Young University, Provo, Utah, United States of America; 2 Mechanical Engineering Department, Brigham Young University, Provo, Utah, United States of America; 3 Athlete High Performance Department, United States Figure Skating Association, Colorado Springs, CO, United States of America; Baylor College of Medicine, UNITED STATES

## Abstract

Advancements in wearable technology have facilitated performance monitoring in a number of sports. Figure skating may also benefit from this technology, but the inherent movements present some unique challenges. The purpose of this study was to evaluate the feasibility of using an inertial measurement unit (IMU) to monitor three aspects of figure skating jumping performance: jump count, jump height, and rotation speed. Seven competitive figure skaters, outfitted with a waist-mounted IMU, performed a total of 59 isolated multi-revolution jumps and their competition routines, which consisted of 41 multi-revolution jumps along with spins, footwork, and other skills. The isolated jumps were used to develop a jump identification algorithm, which was tested on the competition routines. Four algorithms to estimate jump height from flight time were then evaluated using calibrated video as a gold standard. The identification algorithm counted 39 of the 41 program jumps correctly, with one false positive. Flight time and jump height errors under 7% and 15% respectively were found using a peak-to-peak scaling algorithm. Rotation speeds up to 1,500°/s were noted, with peak speeds occurring just over halfway between takeoff and landing. Overall, jump monitoring via IMUs may be an efficient aid for figure skaters training multi-revolution jumps.

## 1. Introduction

Figure skating has evolved throughout its history from an artform to an athletic sport. Notably, in 1990 the discipline of figures, to which the sport owes its name, was eliminated from competitions. This event accelerated an already ongoing shift in training time from figures to freestyle, and specifically to jumping. Judging criteria also evolved to reward more technically demanding programs. Today’s competitive skaters train extensively, engaging in several hours daily of both on-ice and off-ice workouts. The number of jumps that elite skaters perform each day has been roughly estimated at between 50 and 100 [1], but to our knowledge has not been formally tracked. With a potential link between increased jumping and increased injury rates [2–8], there is a need to track jump training volume.

Elite figure skaters often face a potential conflict between performance improvement and injury prevention. Numerous jump repetitions are performed to learn and hone technique, yet repetitive impacts from jump landings are likely linked to chronic overuse injuries. For many skaters and coaches, short-term desires for performance improvement may supersede any concerns of long-term injury risks. This dilemma is not unique to figure skating. Many sports have increased monitoring and even regulations in an attempt to balance injuries and performance. For example, in baseball, pitch count limits have been instituted [9, 10], while in American football there has been an increase in monitoring head impacts related to concussions [11]. Advancements in wearable technology have increased the ability to measure and monitor various parameters related to both injury risk and performance in a variety of sports. This has the potential to increase understanding of risk factors, raise awareness of training volume, and increase training efficiency. Figure skating may also benefit from this technology.

Existing wearable technology, in the form of an accelerometer and gyroscope (called an inertial measurement unit, or IMU), could be used to monitor three important parameters for figure skating: jump count, jump height, and rotation speed. Jump count could be used to document, monitor, and regulate training volume while jump height could be used to measure jumping performance as well as monitor fatigue or performance degradation. Rotation speed could similarly be used as a performance feedback aid and a measure of performance degradation. The combination of the three parameters has the potential to help skaters train more efficiently, which may be the key to balancing performance and injury conflicts. However, there are a number of challenges in deriving these quantities from IMU signals in figure skating.

These three quantities depend on the ability to identify a jump from an accelerometer signal. For jumping in general, acceleration peaks are found near take-off and landing events. Yet, figure skating performances often contain footwork and other movements that could contain acceleration peaks that mimic those from jumps. Determining optimal peak detection tolerances is therefore important in avoiding false positives. Once a jump is identified, jump height can be calculated from flight time using a Newtonian projectile motion relationship (see Methods 2.3.2). A number of accelerometer-based methods to determine flight time have been applied to vertical jumping, with good accuracy [12–14]. Additional challenges are inherent in figure skating due to the rotation that accompanies jumps as well as potential differences between take-off and landing positions.

The purpose of this study was to determine the feasibility and initial accuracy of using an IMU to measure multi-revolution jump count, jump height, and rotation speed in figure skating. We theorized that the combined use of an accelerometer and gyroscope could successfully capture the number of multi-revolution jumps performed despite the presence of numerous confounding movements inherent in this sport.

## 2. Methods

### 2.1 Participants

Seven healthy competitive figure skaters between the ages of 12 and 27 years participated in the study (6 F, 1 M). Skaters’ competition levels ranged from Juvenile to Senior (as determined by their participation in U.S. Figure Skating sanctioned events), with all skaters able to perform at least double revolution jumps. All skaters were volunteers and they and their parents (when necessary) signed assent/consent forms approved by the Brigham Young University Institutional Review Board. Subject mean ± standard deviation height and mass were 1.57±0.06 m and 49.2±6.3 kg.

### 2.2 Data collection

Data collection consisted of two parts, the order of which was determined by the skater’s preference: isolated jumps and a competition routine. The isolated jumps were used as a training set for the jump-finding algorithm and as the primary analysis for the jump height calculations. The competition routine, which included jumps, spins, and footwork, was used as a testing set to evaluate potential acceleration signals that could confound jump identification. Jumps were classified as either successful or unsuccessful. Successful jumps were landed cleanly on one foot, while falls, under-rotated jumps, and two-footed landings were considered unsuccessful. Classification was performed from video by two experienced skaters.

Prior to collection, each skater was outfitted with an IMU (Opal model, APDM, Inc., Portland OR, USA), affixed to the lower back at approximately the L4-L5 spinal level with elastic therapeutic tape (KT Health, LLC, American Fork UT, USA). The IMU axes were aligned with the anatomical cardinal planes. As video recording was used for jump height validation, a high contrast color elastic strap was wrapped around the waist at approximately the same level as the IMU and used as a visual surrogate for the skater’s center of mass. A location on the ice was marked for the isolated jumps. A high-speed camera (Sony nex-fs700U with a Canon EFS 17–55 mm lens), mounted on a tripod, was positioned approximately 8 meters from this location. A range pole was used as a distance calibration reference. Snapshots of the pole were collected with it held on the jump location as well as two other depths: approximately 0.5 m in front of and behind the location. During jump testing, the closest of the three reference positions to the jump mid-flight location was noted.

**Fig 1:**
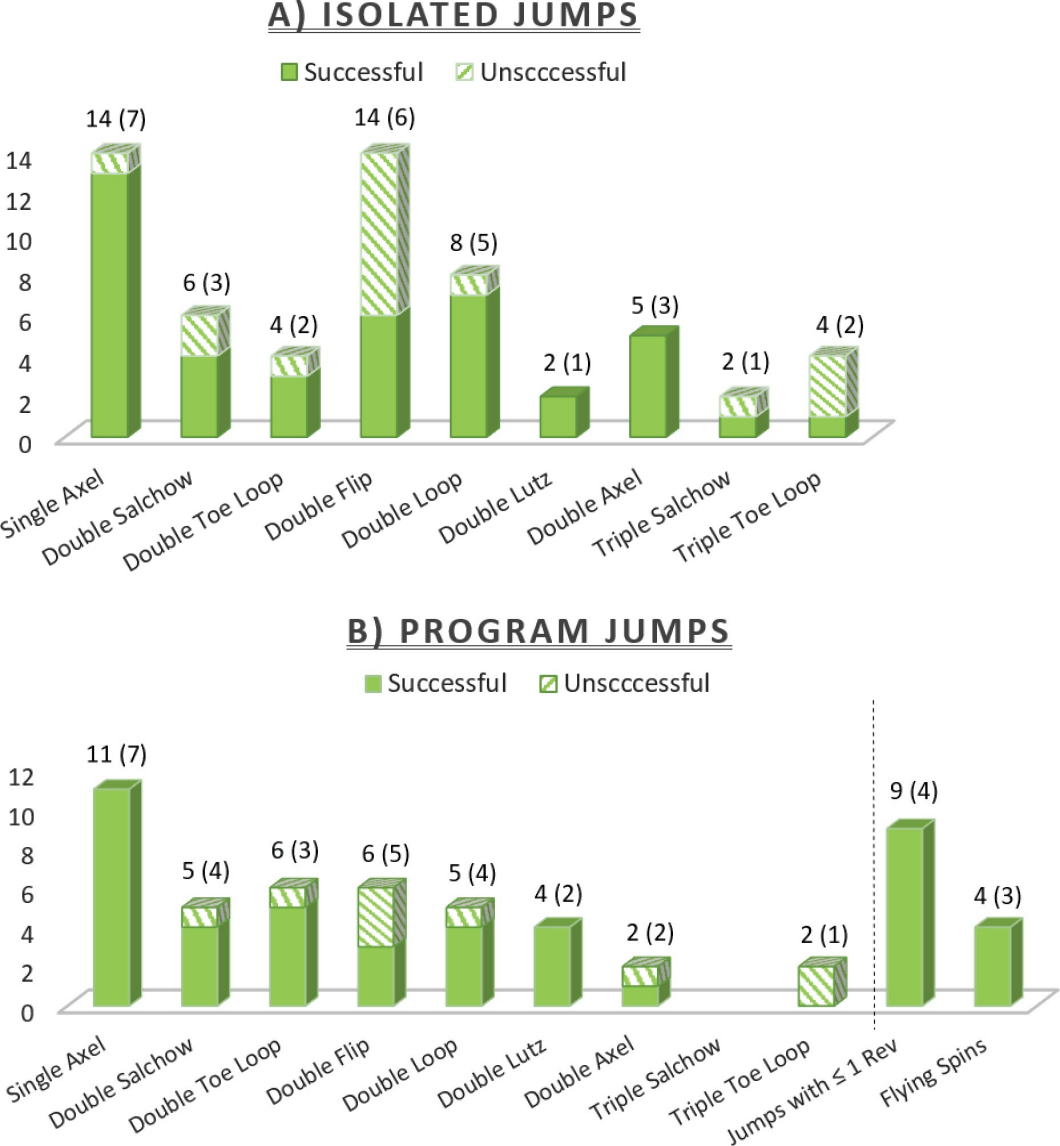
Tally of all jump types performed. The numbers at the top of each stacked bar represent the total number of jumps performed and number of skaters who contributed to that tally in parentheses. All jumps were marked as being landed successfully or unsuccessfully, the latter designation given to falls, under-rotations, and two-footed landings. For the isolated jumps (A), a total of 59 multi-revolution jumps were recorded from seven skaters. Forty-two (71%) of the jumps were successfully landed and 17 (29%) were unsuccessful. From the competition routines (B), a total of 41 multi-revolution jumps were recorded from the seven skaters. Thirty-two (78%) of the jumps were successfully landed and nine (22%) were unsuccessful. In addition, nine jumps of one rotation or less and 4 flying spins were tallied.

Each skater performed between six and ten isolated multi-revolution jumps. These consisted primarily of single axels (1.5 revolutions) and various double revolution jumps, with a few of the simpler triple revolution jumps (Fig 1A). The numbers and types of jumps performed by each skater were not controlled, as the goal was simply to achieve a sufficiently broad data set to perform a group analysis without overburdening each skater. Competition routines lasted between 2.0 and 3.5 minutes, depending on the level of the skater, and contained between 4 and 11 jumps (Fig 1B) interspersed with other movements. IMU data was recorded for both the isolated jumps and the competition routine at 128 frames/s. Video was recorded at 240 frames/s with a resolution of 1920 by 1080 pixels for the isolated jumps. Competition routines were recorded with a cell phone camera, which was used only as a reference.

### 2.3 Data analysis

The IMU data was analysed using custom MATLAB software (Mathworks, Inc., Natick MA, USA). All accelerometer signals were processed with a low pass filtered (6th order Butterworth with 10Hz cutoff) prior to analysis. MATLAB’s findpeaks() function was then used to identify jumps and implement jump height algorithms. Video analysis was performed using Dartfish software (Dartfish USA, Inc. Alpharetta GA, USA).

#### 2.3.1 Jump identification

**Fig 2:**
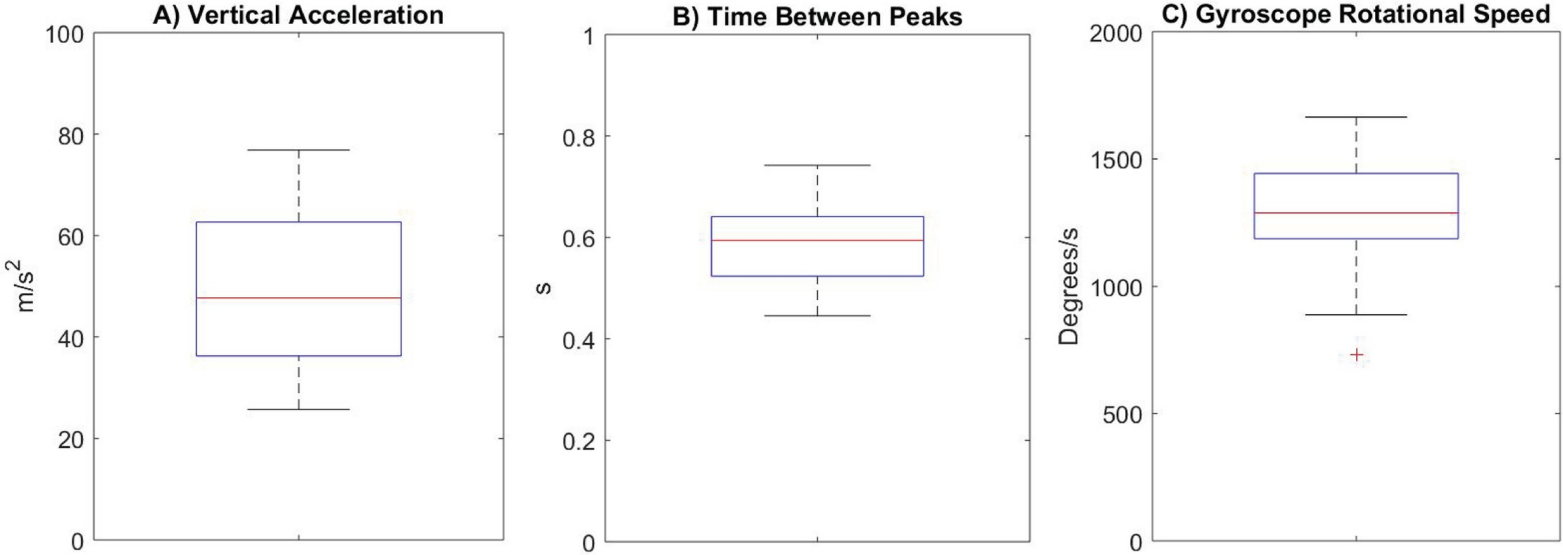
Boxplots characterizing all isolated jumps. A) Peak vertical accelerations during take-off and landing; B) Time between peak accelerations; and C) Peak rotation speed between peak accelerations (as measured by the gyroscope). Boxplots are median and quartiles with range whiskers, outlier represented by red pluses.

Video and IMU data were matched by manually identifying the two acceleration peaks (from the IMU data) that corresponded with the beginning and end of each jump (from the video). These acceleration peak magnitudes were extracted from the IMU data along with the time between peaks and the peak rotational velocity occurring between them (Fig 2). The algorithm for automated jump detection was developed from this manual identification, using a three-step process. The appropriate IMU signal thresholds to be used in each step were determined from the isolated jumps, with some additional tolerances added to slightly broaden the characterization. In order to be counted, a jump had to include the following:

1. Two acceleration peaks (take-off and landing), both greater than 25 m/s^2^_._
2. Acceleration peaks separated by 0.3 s to 0.85 s.
3. A rotation peak greater than 688 deg/s (12 rad/s) between the two acceleration peaks.

Following characterization of the isolated jumps, the three-part algorithm, with the above thresholds, was applied to the competition routines to determine its effectiveness in identifying multi-revolution jumps while avoiding false positives. The number of jumps identified by the IMU algorithm, along with the number of correct jumps (tallied from reference video) were quantified at each algorithm step.

#### 2.3.2 Jump height

**Fig 3:**
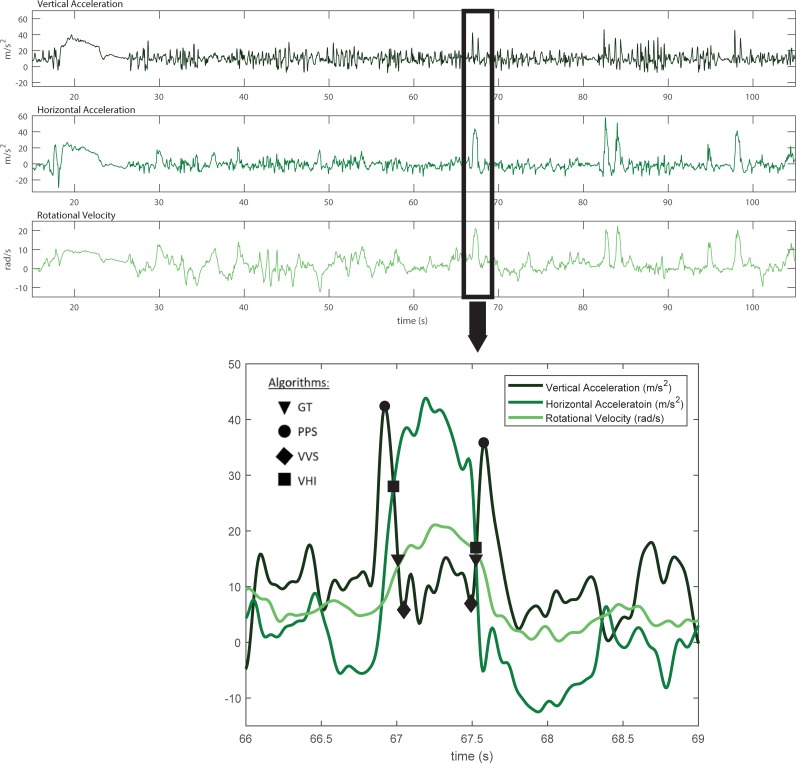
Example IMU signals. The first half of a roughly 3.5 minute long Novice level men’s competition routine is shown (Top). The top signal represents vertical acceleration, the middle represents radial acceleration, and the bottom represents rotation around the vertical axis (from the gyroscope). A three-second sub-section surrounding a double flip jump is highlighted and expanded (bottom). Each of the three signals are plotted on the same axis to show their interactions. Take-off and landing events from the four algorithms are also shown using symbols. Take-off events are on the left, just following the first peak, while landing events are on the right, just prior to the last peak.

IMU based jump height estimation was based on determining flight time (t). Assuming that the center of mass height at take-off and landing are the same and that air resistance is negligible, jump height can be determined from the physics of a free falling body: Height = gt^2^/8. However, peak accelerations in most jumps typically occur slightly before the instant of take-off and slightly after the instant of landing; therefore jump height estimation focused on potential algorithms to either determine take-off and landing events in the accelerometer signal, or adjust the time between accelerometer peaks. Four potential flight time algorithms were considered (Fig 3):

1. *Gravitational threshold (GT)* (Fig 3B, triangles): Take off was marked as the time following the first peak when acceleration dropped below 1.5*g. Landing was marked as the time when it again rose above 1.5*g just before the second peak. This algorithm was adapted from Monnet et al. [13].
2. *Peak to peak scaling (PPS)* (Fig 3B, circles): The time between accelerometer peaks was multiplied by a scale factor. This scale factor was calculated by dividing the mean peak to peak time (from IMU) by the mean flight time (from video). The resulting scale factor was 0.761.
3. *Valley to valley scaling (VVS)* (Fig 3B, diamonds): The time between the first valley following the take-off peak and the last valley before the landing peak was multiplied by a scale factor. This scale factor was calculated by dividing the mean valley to valley time (from IMU) by the mean flight time (from video). The resulting scale factor was 0.887.
4. *Vertical / horizontal acceleration intersection (VHI)* (Fig 3B, squares): Take-off and landing were marked at the intersection of the vertical and horizontal accelerations (just after vertical take-off peak and just before vertical landing peak).

**Fig 4:**
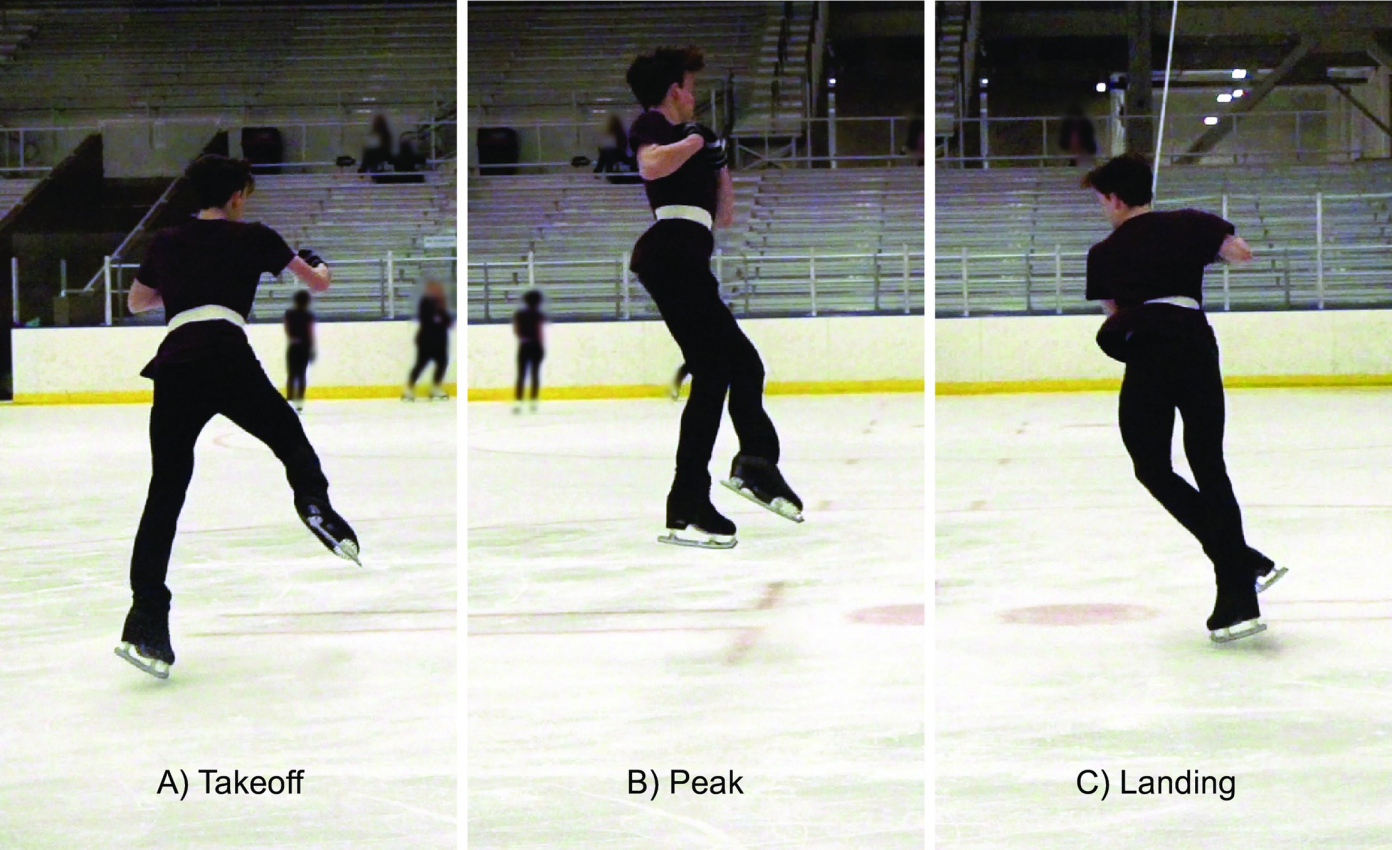
Video identification of jumping events. Snapshots of the take-off (A), peak (B), and landing (C) events from a subject performing an isolated double axel jump. The high contrast waistband is shown, from which spatial jump height measurements were taken. The individual in this picture has given written informed consent to publish this photograph.

Flight time and jump height were obtained from the isolated jump videos and used to determine algorithm accuracy (Fig 4). First, the events of take-off (the frame when the toe of the take-off leg first lifted off the ground) and landing (the frame when the landing toe first touched the ground) were marked. The time between the two events was considered the gold standard for flight time. Next, the positions of the high contrast waistband were measured at take-off, peak height, and landing positions. The snapshot of the ranging pole, in the appropriate reference position, was used to convert pixels to centimeters. Video analysis was performed by two separate researchers, with values averaged between them.

The temporal information from the video was considered more accurate than the spatial information, so accuracy of the algorithm was first assessed by comparing IMU algorithm based flight times to video based flight times using mean absolute errors (MAEs). Jump heights derived from these flight times were similarly compared using MAEs. The spatial video analysis was used primarily to determine whether there were any differences between take-off and landing height positions. To do this, ascent height (vertical position at peak minus takeoff) was compared to descent height (peak minus landing).

#### 2.3.3 Rotation speed

Analysis of rotation speed focused on tabulating peak rotation speeds along with the timing of these peaks relative to take-off and landing events. This was done across all isolated and competition routine jumps. Peak rotation speeds were tabulated for all multi-revolution jumps, as well as separated between double and triple jumps.

## 3. Results

### 3.1 Jump identification

**Fig 5:**
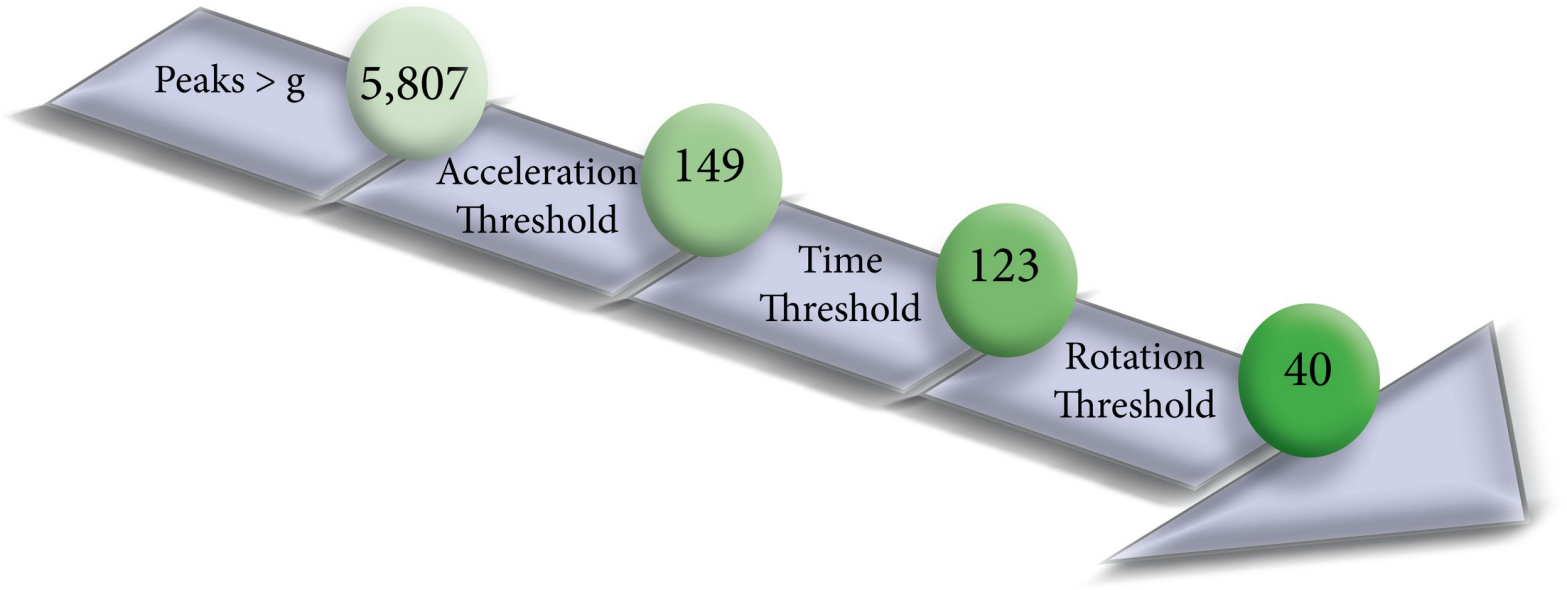
Jump identification flow chart. This shows the effect of each step of the jump identification algorithm on the competition routines. A total of 5,807 peak pairs were present, indicating potential jumps. This was reduced to 40 jumps with the three-step algorithm. There were 41 total multi-revolution jumps performed, 39 of these jumps were captured by the algorithm (missing 2), and one ≤ one revolution jump was captured.

Applying the three-step jump identification algorithm (developed on the isolated jumps) to the competition routines successfully captured 39 of the 41 multi-revolution jumps. The two missed jumps were a double loop that was the second jump in a two-jump combination (no steps between jumps), and a double flip in which the skater fell on the landing. One of the single revolution jumps also met the criteria. This was a single loop that was originally intended to be a double, but the rotation was aborted in the air (i.e. popped). The algorithm did not capture the other nine ≤ one revolution jumps. None of the flying spins, footwork, or connecting movements were captured (Fig 5).

### 3.2 Jump height

**Fig 6:**
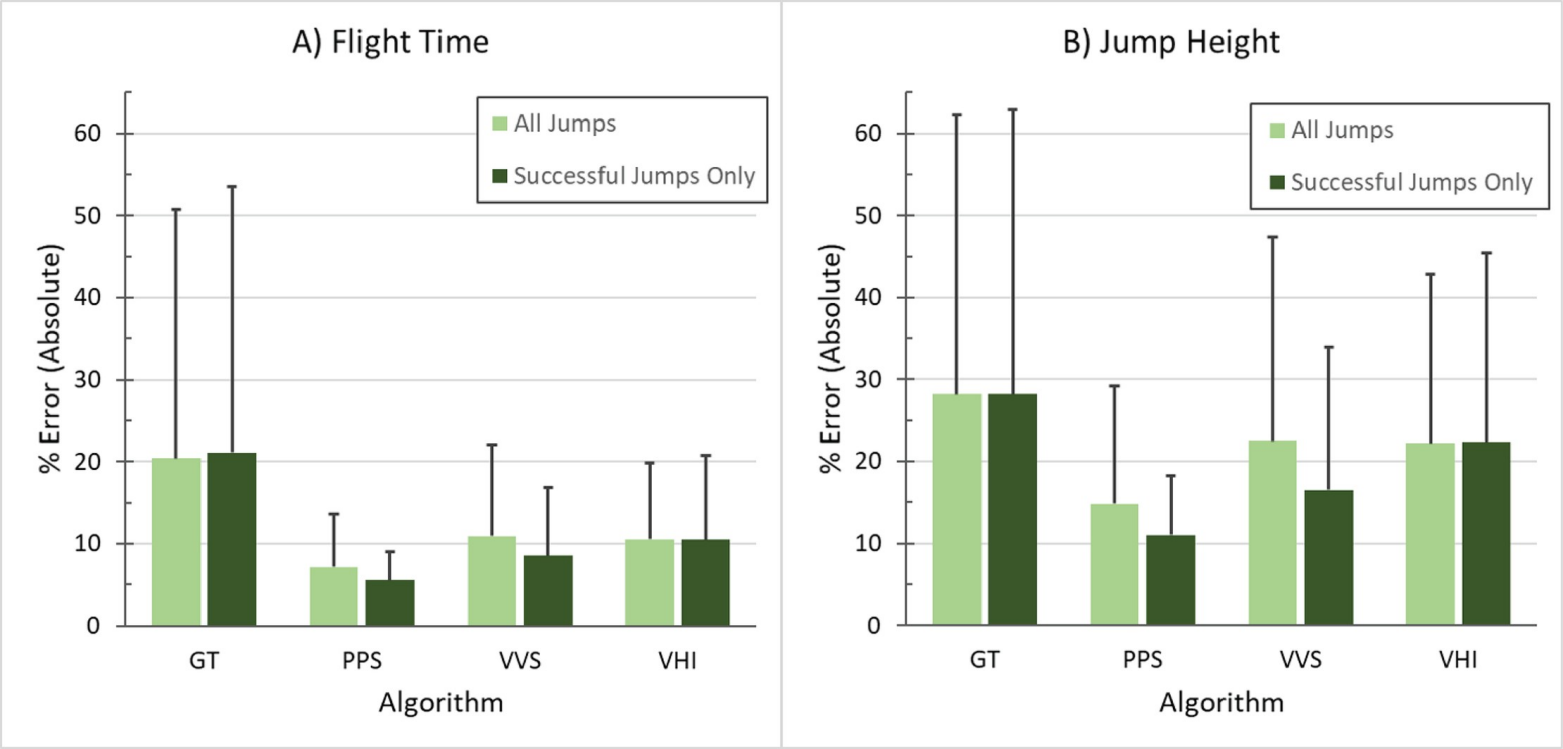
Algorithm accuracy expressed as % error. All four algorithms were tested on the isolated jumps to determine accuracy in flight time (A) and jump height (B).

PPS was the most accurate method, with MAEs of 0.03 s in flight time or 3.3 cm in derived jump height (Table 1, Fig 6). The worst performing method was GT, which had MAEs of 0.098 s in flight time and 7.81 cm in jump height. VHI tended to slightly overestimate jump height, while GT tended to underestimate it (PPS and VVS were scaled from the means and thus inherently matched mean values). When algorithms were applied only to the successfully landed jumps, accuracy improved slightly for PPS and VVS (but not GT and VHI), with PPS MAEs reaching 0.024 s in flight time and 2.62 cm in jump height.

**Table 1:** Flight time and jump height.

Method	All Isolated Jumps		Successful Jumps Only	
	Flight time (s)	Jump Height (cm)	Flight time (s)	Jump Height (cm)
**Video**	0.44 ± .060	24.4 ± 6.5	0.44 ± .058	24.4 ± 6.2
***Errors compared to video*:**				
**GT**	0.098 ± 0.154	7.81 ± 10.8	0.103 ± 0.165	8.00 ± 11.4
**PPS**	0.031 ± 0.025	3.33 ± 2.75	0.024 ± 0.015	2.62 ± 1.72
**VVS**	0.047 ± 0.046	7.81 ± 10.83	0.040 ± 0.041	4.36 ± 4.84
**VHI**	0.165 ± 0.053	4.87 ± 3.87	0.167 ± 0.056	4.85 ± 4.13

Flight time and derived jump height from video, followed by mean absolute errors (MAEs) from the four IMU-based algorithms (as compared to video). MAEs were calculated as the absolute differences between each method and the gold standard video (expressed as means ± standard deviations). Evaluation consisted of all isolated jumps followed by only the successfully landed jumps.

**Fig 7:**
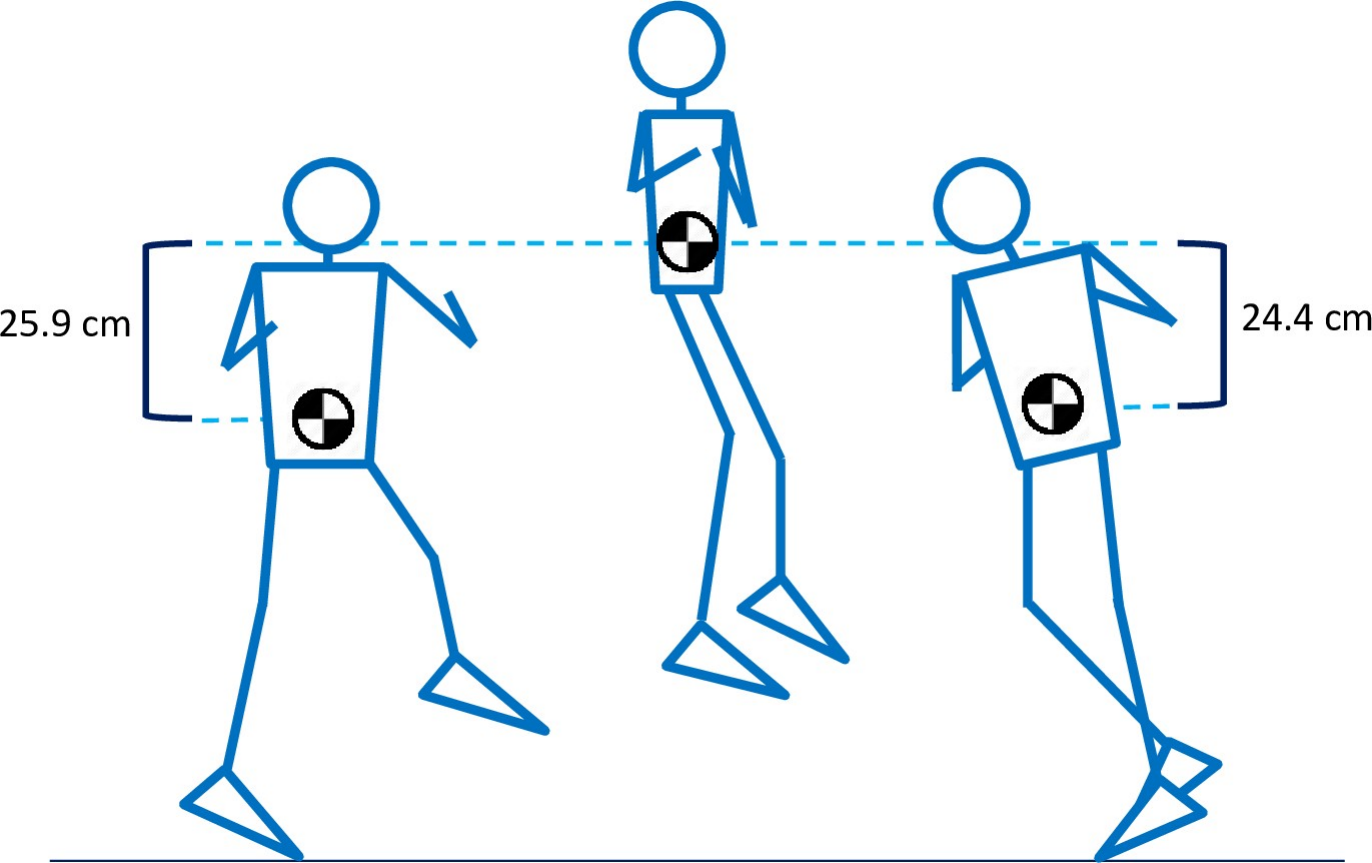
Spatial analysis of isolated video jump heights. Ascent height was calculated as the difference between vertical positions at peak and take-off events. Descent height was similarly the difference between peak and landing events. Landing position was on average 1.53 cm higher than take-off position.

Jump height measured from spatial video analysis was on average 1.5 cm higher (25.9 ± 7.1 cm) than that derived from video flight time (24.4 ± 6.5). Spatially measured landing positions were on average 1.5 cm (± 4.2 cm) higher than take-off positions (Fig 7).

### 3.3 Rotation speed

Peak rotation speed across all jumps ranged from 889 to 1,665 deg/s, with a mean of 1,289 deg/s (see also Fig 1C). Separating double and triple jumps, the mean speed for the doubles was 1,273 deg/s and the mean speed for the triples was 1,465 deg/s. The peak rotation speed occurred 64 ± 16% (mean ± SD) of the way between the two acceleration peaks.

## 4. Discussion

The purpose of this study was to determine the feasibility of using an IMU to characterize multi-revolution jumping performance in the sport of figure skating. We performed a systematic analysis on 59 isolated training jumps as well as competition routines containing 41 multi-revolution jumps and confounders such as spins, footwork, and other skills. The results are promising for jump identification and rotation speed quantification, but some accuracy hurdles remain for accurate jump height estimation.

### 4.1 Jump identification

Out-of-sample testing on the competition routines showed the potential for accurate multi-revolution jump detection despite the presence of numerous confounding movements. These non-jump movements also demonstrated the importance of all three steps in the algorithm. For example, a number of movements met the first two algorithm criteria. Jump count was reduced from 123 to 40 only after applying a rotational requirement. There were only two false negatives. One was a fall in which the skater did not have a strong landing impact peak due to off-axis positioning and continued rotation after landing contact. The other was the second jump in a two-jump combination, having a small take-off acceleration (and low jump height). There was also one false positive, a jump that was meant to be a double jump but was aborted in mid-flight. This jump displayed initial acceleration and rotation characteristics more similar to a double jump than a single jump. All other single jumps were excluded in our algorithm due either to insufficient acceleration or insufficient rotation, and usually both.

We targeted jumps greater than one revolution as these are generally considered more dangerous than single jumps, perhaps due to reduced landing preparation time [15]. However, studies on impacts and injury development are still lacking; the proposed device could be a first step towards empirically determining these relationships. If the supposed link between jumps and injuries is related primarily to peak accelerations, it may be that single jumps and other movements could also contribute to the cumulative toll that jump impacts apply to the body. Many of the non-jump movements, such as footwork and flying spins, had accelerations similar to single jumps, and sometimes doubles. Lowering thresholds to capture single jumps is certainly possible, but would also capture many of these other movements. More research is needed to determine the extent to which single jumps should be monitored. Our dataset also consisted of intermediate to upper level skaters, which specifically train for double and triple jumps; it is possible that lower level skaters may have greater distinction between single jumps and other skills. Subject specific tuning of algorithm parameters may address these issues, but would require a larger sample to fully implement and evaluate.

### 4.2 Jump height

PPS was the most accurate algorithm for calculating jump height. However, PPS had a slight inherent advantage as its scaling factor was tuned directly to the data (note that VVS was also tuned to the data and it performed poorly). GT performed the worst but may have some future potential. Its poor performance was due primarily to fluctuations in vertical acceleration during air time, resulting in delays in crossing the 1.5g threshold in some subjects. Thirteen of the 59 isolated jumps had obvious, large delays in threshold crossing. If these 13 are removed, accuracy improves to rival that of PPS, with mean errors of 5.7% in flight time and 11.5% in jump height (compared to 5.5% and 11.1% for PPS on successful jumps). Some of the acceleration variability during air time may be due to off-axis rotations, which could be improved with a more complex algorithm that incorporates orientation estimates. For further discussion of this specific algorithm limitation, see 4.4 below. VHI utilized the crossing of the vertical and radial acceleration signals, which we originally thought could have some physiological relevance in signifying a transition from upwards linear acceleration to rotational motion. However, the variability in the signals makes this crossing an unreliable indicator of take-off or landing. It is possible that thresholds in radial acceleration or gyroscope signals could be used to indicate how take-off and landing events relate to rotation—this could be jump type specific and would require a larger data set to fully evaluate.

While temporal information from the video analysis was considered a priori to be more accurate than spatial information, both appeared to be in general agreement, allowing us to draw some preliminary conclusions regarding future jump height accuracy. Spatial video analysis was used primarily to look for differences between take-off and landing positions. We found that landing was on average 1.5 cm higher than take-off (i.e. subjects were more extended at landing)—this would result in a slightly shorter flight time (compared to landing at an equal height) and therefore a slight underestimation of true jump height when derived from flight time. Using the mean values for flight time (0.44s) and height difference (1.5 cm), this should result in a mean underestimation of approximately 1.0 cm (24.47 cm—23.7 cm). This is close to the spatially measured jump height, which was on average 1.5 cm higher than that determined from flight time. It may therefore be possible to improve overall jump height accuracy by incorporating a fixed, or subject specific, height difference into the algorithm. Alternately, it may be sufficient to simply focus training programs around within-subject changes in flight time itself, rather than trying to derive accurate measures of jump height. For many skaters, focusing only on these changes may still be extremely beneficial in identifying fatigue onset or documenting performance improvements.

### 4.3 Rotation speed

Rotation speed may be the most desirable monitoring parameter for increasing skating jump performance. To accomplish more difficult jumps, skaters can either increase jump height or increase rotation speed. It is generally easier to lower rotational inertia by adjusting air position than it is to gain jumping height, as evidenced by research showing no differences in jump height between double and triple jumps [15]. A few previous studies have measured peak rotation speeds derived from video analysis [15–17]. Our peak rotation speeds from the IMU gyroscope compare favorably with these studies. In addition to peak rotation speed, other aspects of the rotation speed/ time profile may be beneficial to training; for example, in determining how quickly skaters achieve peak angular velocity. In our study, peak rotation speeds occurred on average just over halfway (64%) between the take-off and landing acceleration peaks. In order to complete more rotations, this may need to occur earlier in the jump.

### 4.4 Limitations and future opportunities

This was a feasibility study, and as such, we employed a relatively small sample size. There were also some discrepancies between the difficulty of the isolated jumps and the jumps contained in the competition routines, with slightly lower difficulty in the competition routines because skaters opted out of performing many of their harder jumps within the constraints of the routine. However, these differences had no detrimental effect on the jump count algorithm itself, which performed well across all competition routines. The main advantage of a larger sample size would be the ability to perform additional analyses; for example, distinguishing jump types (e.g. edge jumps from toe jumps) or investigating subject-specific threshold tuning. For example, with a larger sample size machine learning methods could be used to help classify jumps within competition routines.

Additional complexity could also be added to the algorithms to overcome potential drift and orientation hurdles. Gyroscopes have inherent drift, which we did not control for in our short-duration testing, but this may be a factor in longer duration monitoring. We also utilized the IMU sensor components independently, aligning the IMU axes to the cardinal body planes. Axis misalignment errors are therefore possible due both to placement and changes in body posture. Two approaches to overcome misalignment errors have been used in other studies. The first simply combines the individual acceleration components into a single resultant acceleration [18], but this does not appear to be feasible in skating due primarily to high radial accelerations when rotating. The second relies on a sensor fusion algorithm to calculate orientation and transform the sensor to body specific axes [19, 20]. Orientation accuracy has not been tested in figure skating, and our positive results suggest that only minor improvements are likely with the additional complexity. A few instances warrant note, however. Two of the isolated double axel jumps contained an extra acceleration peak just after take-off. This was caused by radial acceleration that bled into the vertical component because the skater had substantial off-axis body lean. This only occurred twice, but could negatively affect jump identification in some skaters. Additionally, some of the flying spins have substantial changes in body posture that could similarly confound jump identification.

## 5. Conclusions

In this study we developed a prototype jump monitor for figure skating. Overall, our results suggest that accurate identification of multi-revolution jumps and quantification of rotation speeds can be accomplished using a single waist-mounted IMU. Further algorithm development could increase jump height estimation accuracy. A fully integrated jump monitor which incorporates these capabilities may increase training efficiency and help skaters and coaches balance injury and performance conflicts.
